# Principles of the Mechanism for Epimuscular Myofascial Loads Leading to Non-uniform Strain Distributions Along Muscle Fiber Direction: Finite Element Modeling

**DOI:** 10.3389/fphys.2020.00789

**Published:** 2020-07-03

**Authors:** Uluç Pamuk, Alican Onur Cankaya, Can A. Yucesoy

**Affiliations:** Biomechanica Laboratory, Institute of Biomedical Engineering, Boğaziçi University, Istanbul, Turkey

**Keywords:** finite element model, epimuscular myofascial loads, non-uniform length change, muscle fiber direction, extracellular matrix, intermuscular interactions

## Abstract

Sarcomere lengths and their changes are key determinants of muscle active force production. Recent studies indicate inhomogeneity of sarcomere lengths within the muscle. Studies utilizing magnetic resonance imaging (MRI) analyses for quantifying local muscle tissue strains and diffusion tensor imaging (DTI) analyses allowing for determination of their components along muscle fascicles show that those length changes can be non-uniform. Specifically, two questions arise regarding the muscle’s length change heterogeneities along the muscle fiber direction: (1) How can a passively lengthened muscle show shortened regions? (2) How can an isometric contracting muscle show lengthened parts? Using finite element modeling and studying principles of the mechanism of strain heterogeneity along the muscle fiber direction, the aim was to test the following hypothesis: epimuscular myofascial loads can lead locally to strains opposing those elsewhere within the muscle that are determined by the globally imposed conditions. The geometry of the model was defined by the contour of a longitudinal slice of the rat extensor digitorum longus (EDL) muscle belly. Three models were studied: (1) isolated muscle (muscle modeled fully isolated from its surroundings) and models aiming at representing the principles of a muscle in its *in vivo* context including (2) extramuscularly connected muscle (muscle’s connections to non-muscular structures are modeled exclusively) and (3) epimuscularly connected muscle (additionally muscle’s connections to neighboring muscle are modeled). Three cases were studied: passive isometric muscle with imposed relative position change (Case I), passive lengthened muscle (Case II), and active isometric muscle with imposed relative position change (Case III). The findings indicated non-uniform strains for all models except for zero strain in model (1) in Case I, but models (2) and (3) also showed strains opposing the imposed effect. Case I: model (3) showed shortened and lengthened sections (up to 35.3%), caused exclusively by imposed relative position change. Case II: models (2) and (3) showed shortened sections (up to 12.7 and 19.5%, respectively) in addition to lengthened sections. Case III: models (2) and (3) showed lengthened sections (up to 5 and 23.4%, respectively) in addition to shortened sections. These effects get more pronounced with stiffer epimuscular connections. Assessments of forces exerted on the muscle by the epimuscular connections showed that such strain heterogeneities are ascribed to epimuscular myofascial loads determined by muscle relative position changes.

## Introduction

Muscle has a multi-scale composite structure (Huijing, 1999): its fundamental force producing units, sarcomeres, are connected to each other serially at z-discs (Rowe, 1971), making up myofibrils, while adjacent myofibrils are interconnected and are also connected sequentially to laminin, the basal lamina, and endomysium via multimolecular and costameric proteins (Pardo et al., 1983; Danowski et al., 1992; Berthier and Blaineau, 1997), which forms a three-dimensional structure in which muscle fibers operate (Trotter and Purslow, 1992). Therefore, it is representative to consider skeletal muscle in a mechanically linked bi-domain concept of activatable muscle fibers and the extracellular matrix (ECM) (Yucesoy et al., 2002). Intramuscularly, this concept allows mechanical interaction of these domains such that local length changes along the muscle fiber direction is determined by the mechanical equilibrium affected by forces (*myofascial loads*) exerted by the ECM and other muscle fibers. The role of the ECM in limiting shortening of sarcomeres in an activated muscle fiber (Street, 1983) and in limiting elongation of sarcomeres in passively stretched muscle fibers (Street, 1983) was shown.

Additionally, all along the muscle belly, the ECM is continuous with structures outside the muscle: collagen reinforced connective tissue structures supporting the neurovascular tracts (i.e., the tissues containing nerves and blood vessels), compartmental boundaries, intermuscular septa, interosseal membrane, and the bone are in continuity and there is connectivity between the epimysia of adjacent muscles. This connective tissue integrity (for pictures, see, e.g., Huijing and Baan, 2001; Huijing et al., 2003; Yucesoy et al., 2006; Stecco et al., 2009; Willard et al., 2012; Wilke et al., 2016) allows for transmission of forces to and from the muscle through non-tendinous pathways. *Extramuscular connections* are defined to represent muscle’s connections to non-muscular structures exclusively. *Epimuscular connections* are defined to represent muscle’s connections to both surrounding non-muscular and muscular structures. Therefore, extramuscular and epimuscular myofascial loads can also affect the mechanical equilibrium determining local length changes along the muscle fiber direction. The mechanical interactions of the muscle with its surrounding muscular and non-muscular structures together are referred to as epimuscular myofascial force transmission (EMFT) (Huijing, 2009; Yucesoy, 2010).

Previously, effects of such myofascial loads have been shown to cause, e.g., proximo-distal muscle force differences and mechanical condition dependent muscle length–force characteristics in animal studies as well as length change inhomogeneities along muscle fascicles in accompanying finite element modeling studies (Yucesoy et al., 2003a, b). Moreover, *in vivo* human studies using elastography (Ateş et al., 2018) and magnetic resonance imaging (MRI) analyses (Huijing et al., 2011) have shown that knee joint angle change imposed at restrained ankle angle causes non-uniform changes in the local stiffness and local length distributions of soleus muscle, respectively. Further studies combining diffusion tensor imaging (DTI) tractography to determine directions of muscle fascicles combined with MRI deformation analyses underlined the possibility of non-uniform deformations along muscle fascicles occurring *in vivo* under both passive and active conditions (Pamuk et al., 2016; Karakuzu et al., 2017). Yet, mechanical principles of the mechanism behind the findings reported require further elaboration. Specifically, two questions arise from those findings regarding the muscle’s length change heterogeneities along the muscle fiber direction: (1) How can a passively lengthened muscle show shortened regions? (2) How can an isometrically contracting muscle show lengthened parts? By studying principles of the mechanism of strain heterogeneity along the muscle fiber direction, the aim was to test the following hypothesis: epimuscular myofascial loads can lead locally to strains opposing those elsewhere within the muscle that are determined by the globally imposed conditions. For this purpose, finite element modeling was used and muscle fiber direction strains within a truly isolated muscle vs. those of extra- and epimuscularly connected muscles were studied in passive and active conditions.

## Materials and Methods

### Description of the “Linked Fiber-Matrix Mesh Model”

In the linked fiber-matrix mesh (LFMM) model, skeletal muscle is considered explicitly as two separate domains: (1) the intracellular domain and (2) ECM domain. The trans-sarcolemmal attachments are considered as elastic links between the two domains (Yucesoy et al., 2002; Yucesoy and Huijing, 2012).

**FIGURE 1:**
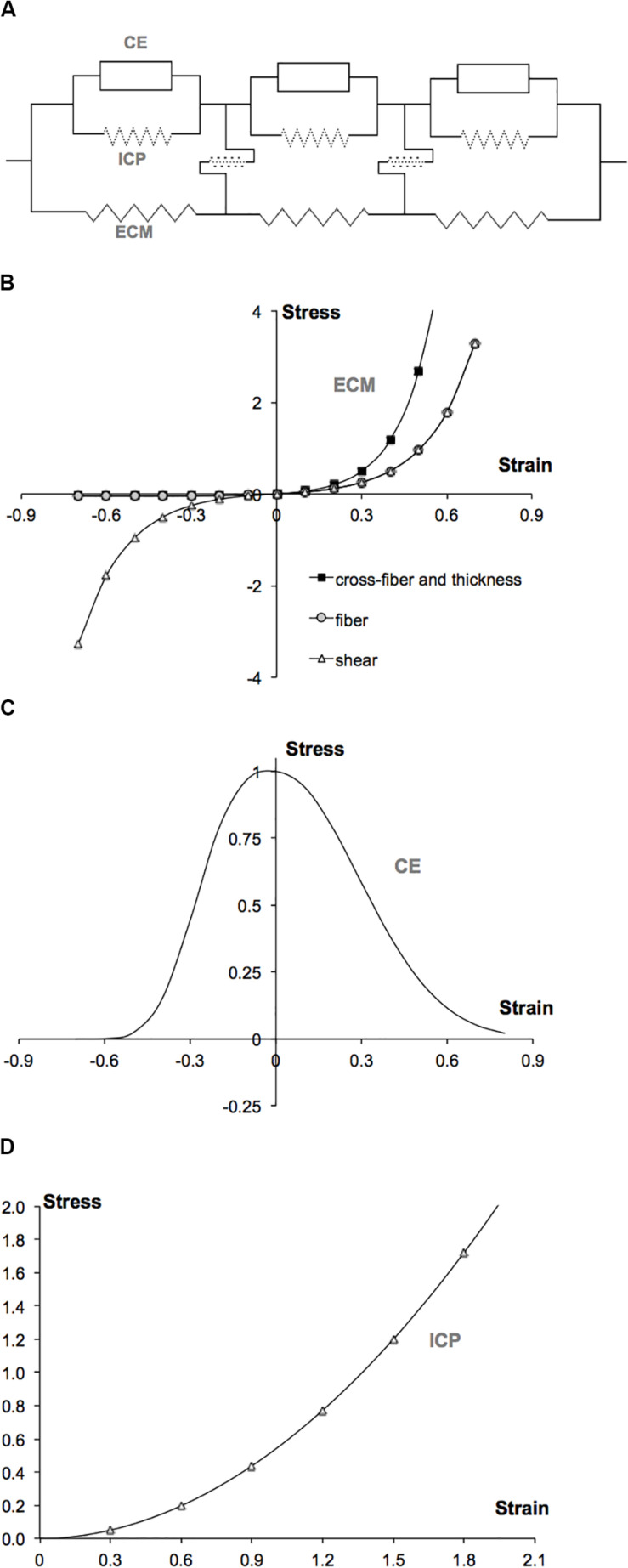
LFMM model concept and plots of constitutive equations defining the muscle modeled. **(A)** 2D schematic representation of an arrangement of muscle elements. The intracellular domain, which is composed of the active contractile elements (CEs) and intracellular passive cytoskeleton (ICP), is linked to the extracellular matrix (ECM) domain, elastically. **(B)** Extracellular matrix element stress–strain relations defined in fiber, cross-fiber, and thickness directions. **(C)** Myofiber element’s active (contractile element) stress–strain relation defined in fiber direction. **(D)** Myofiber element’s intracellular passive (titin) stress–strain relation defined in fiber direction.

Two self-programmed elements were developed and were introduced as user-defined elements into the finite element program ANSYS 12.0. One of these elements (ECM element) represents the collagen reinforced ECM, which includes the basal lamina and connective tissue components such as endomysium, perimysium, and epimysium. A second element models the muscle fibers (myofiber element). Within the biological context, the combined muscle element represents a segment of a bundle of muscle fibers with identical material properties, its connective tissues and the links between them. This is realized as a linked system of ECM and myofiber elements (for a schematic 2D representation of an arrangement of these muscle elements, see Figure 1A).

In the LFMM model, the ECM domain is represented by a mesh of ECM elements (matrix mesh). In the same space, a separate mesh of myofiber elements is built to represent the intracellular domain (fiber mesh). The two meshes are rigidly connected to single layers of elements modeling proximal and distal aponeuroses: a node representing myotendinous connection sites is the common node of all three (ECM, myofiber, and aponeurosis) elements. In contrast, at the intermediate nodes, fiber and matrix meshes are linked elastically to represent the transmembranous attachments of the cytoskeleton and ECM. For these links (the model includes a total of 78 of them: 34 in each of the upper and lower model surfaces) the standard element, COMBIN39 is used from the element library of ANSYS 12.0. This is a two-node spring element, which is set to be uniaxial and have linear high stiffness characteristics representing non-pathological connections between the muscle fibers and the ECM (for an analysis of the effects of stiff or compliant links, see Yucesoy et al., 2002). Note that at the initial length of passive muscle, these links have a length equaling zero.

Both ECM and myofiber elements have eight nodes, linear interpolation functions and a large deformation analysis formulation are applied. A 3D local coordinate system representing the fiber, cross-fiber (normal to the fiber direction), and thickness directions is used. The stress formulation, $\underline{S}$ based on Second Piola–Kirchoff definition, constitutes the derivative of the strain energy density function, W with respect to the Green–Lagrange strain tensor, ${\underline{L}}^{G}$

(1) $$\underline{S}=\frac{dW}{d{\underline{L}}^{G}}$$

#### Extracellular Matrix Element

The strain energy density function mechanically characterizing the ECM includes two parts:

(2) $$W=W_{1}+W_{2}$$

The first part represents the non-linear and anisotropic material properties (Huyghe et al., 1991):

(3) $$W_{1}=W_{ij}\left(\varepsilon_{\text{ij}}\right)$$

where

(4) $$\begin{array}{c} W_{ij}\left(\varepsilon_{\text{ij}}\right)=k.\left(e^{{\text{a}}_{\text{ij}}.\varepsilon_{\text{ij}}}-{\text{a}}_{\text{ij}}.\varepsilon_{\text{ij}}\right)\;\text{for}\varepsilon_{\text{ij}}>0\text{or}\\ W_{ij}\left(\varepsilon_{\text{ij}}\right)=-W_{ij}\left(\left|\varepsilon_{\text{ij}}\right|\right)\;\text{for}\varepsilon_{\text{ij}}<0\text{and}\text{i}\neq \text{j} \end{array}$$

ε_ij_ are the Green-Lagrange strains in the local coordinates. The indices *i* = 1…3 and *j* = 1…3 represent the local cross-fiber, fiber and thickness directions, respectively. a_ij_ and k are constants (Table 1). Note that initial passive stiffness (k) and passive fiber direction stiffness (a_22_) values were estimated by fitting the experimental data by Meijer et al. (1996). Based on the experimental data on dog diaphragm (Strumpf et al., 1993), passive cross-fiber stiffness (a_11_ = a_33_) was taken to be higher than fiber direction stiffness. Identical values are used for fiber and fiber-cross fiber shear stiffness (a_12_ = a_23_ = a_31_). However, in contrast to the non-symmetric stress–strain relationships defined for fiber and cross-fiber directions, a symmetric stress–strain relationship is used for shearing. The resulting stress–strain curves are shown in Figure 1B.

**TABLE 1:** Values and definitions of the model constants.

Constant	Value	Definition
*k*	0.05	Initial passive stiffness (Eq. 4)
*a*_11_	8.0	Passive cross-fiber direction stiffness, *a*_11_ = *a*_33_ (Eq. 4)
*a*_22_	6.0	Passive fiber direction stiffness (Eq. 4)
*a*_12_	6.0	Passive fiber–cross-fiber shear stiffness, *a*_12_ = *a*_23_ = *a*_31_ (Eq. 4)
*S*_*s*_	5.0	Weight factor in the penalty function for the solid volume (Eq. 5)
*S*_*f*_	20.0	Weight factor in the penalty function for the fluid volume (Eq. 5)
*b*_1_	30.0	Coefficient for the stress–strain relation of the contractile elements (Eq. 6)
*b*_2_	–6.0	Coefficient for the stress–strain relation of the contractile elements (Eq. 6)
*b*_3_	1	Coefficient for the stress–strain relation of the contractile elements (Eq. 6)
*t*_1_	0.522	Coefficient for the stress–strain relation of the intracellular passive elements (Eq. 7)
*t*_2_	0.019	Coefficient for the stress–strain relation of the intracellular passive elements (Eq. 7)
*t*_3_	–0.002	Coefficient for the stress–strain relation of the intracellular passive elements (Eq. 7)

The second part includes a penalty function to account for the constancy of muscle volume. The intracellular fluid and solid elastic structures are considered as separate constituents of muscle tissue with different responses to deformation: The intracellular fluid is assumed to have the ability to migrate freely within the cell, whereas the solid elastic structures housing muscle fibers (e.g., basal lamina and endomysium) are restricted in moving as they are being constrained by neighboring cells. Therefore, a penalty function consisting of two parts was used:

(5) $$W_{2}=S_{s}.{\left(I_{3}-1\right)}^{2}+S_{f}.{\left(I_{3}^{avg}-1\right)}^{2}$$

where *I*_3_ is the third invariant (determinant) of the Right Cauchy-Green strain tensor providing a ratio of the deformed local volume over the undeformed local volume for each Gaussian point.

If all *I*_3_’s are kept as unity, the element is considered as solid and the local volumes are conserved. If the weighted mean of all *I*_3_’s per element, ($I_{3}^{avg}$) is kept as unity, the element is considered as a fluid. The penalty parameters *S*_*s*_ (for the solid volume) and *S*_*f*_ (for the fluid volume) allow determining the penalty given for each part. Note that if both *I*_3_’s and $I_{3}^{avg}$’s are unity, the volume is constant. The parameters *S*_*s*_ and *S*_*f*_ (Table 1) chosen after performing specific tests on the ECM element allow a good representation of constancy of muscle volume with optimal numerical efforts: for even very large deformations (e.g., length changes greater than 40%), the maximal deviation from undeformed volume remains below 5%.

#### Myofiber Element

Maximally activated muscle is studied. Sarcomeres within the muscle fibers are assumed to have identical material properties. The force–velocity characteristics are not considered due to the isometric nature of the present work. The total stress for the intracellular domain (σ_22f_) is a Cauchy stress acting in the local fiber direction exclusively and is the sum of the active stress of the contractile elements (σ_22contr_) and the stress due to intracellular passive tension (σ_22icp_).

To define the active length–force characteristics, a piecewise exponential function (Figure 1C) was fit to the experimental data of small rat gastrocnemius medialis (GM) fiber bundles (Zuurbier et al., 1995). This function is scaled such that at optimum length, the fiber direction strain (ε_22_) is zero and the maximal stress value is unity.

(6) $$\begin{array}{c} \sigma_{22contr}\left(\varepsilon_{22}\right)=b_{3}{\text{e}}^{{\text{b}}_{2}\varepsilon_{22}^{2}}\;\text{for}\varepsilon_{22}>0\text{or}\\ \sigma_{22contr}\left(\varepsilon_{22}\right)=b_{3}{\text{e}}^{{\text{b}}_{1}\varepsilon_{22}^{3}}\;\text{for}\varepsilon_{22}<0 \end{array}$$

where *b*_1_, *b*_2_, and *b*_3_ are constants (Table 1).

The source of intracellular passive tension is the intra-sarcomeric cytoskeleton (Trombitás et al., 1995), which is composed of several proteins. In this work, titin is considered to play the dominant role. Experimental tension-sarcomere length data (Trombitás et al., 1995) for a single rabbit skeletal muscle fiber was fitted using a parabolic function (Figure 1D) and scaled to make it compatible to the stress–strain characteristics of the contractile part.

(7) $$\begin{array}{c} \sigma_{22icp}\left(\varepsilon_{22}\right)={\text{t}}_{1}\varepsilon_{22}^{2}+{\text{t}}_{2}\varepsilon_{22}+{\text{t}}_{3}\;\text{and}\\ \sigma_{22icp}\left(\varepsilon_{22}\right)=0\;\text{for}\varepsilon_{22}<0 \end{array}$$

where *t*_1_, *t*_2_, and *t*_3_ are constants (Table 1).

#### Aponeurosis Element

To represent the aponeuroses, a standard 3D, eight-node element HYPER58, from the element library of ANSYS 12.0 is used. This element has a hyperelastic mechanical formulation for which the strain energy density function is defined using the two-parameter Mooney–Rivlin material law:

(8) $$W=a_{10}\left({\bar{I}}_{1}-3\right)+a_{01}\left({\bar{I}}_{2}-3\right)+\frac{\kappa }{2}{\left({\bar{I}}_{3}-1\right)}^{2}$$

where ${\bar{I}}_{i}$*i* = 1…3 are reduced invariants of right-Cauchy strain tensor, *a*_10_and *a*_01_ are Mooney–Rivlin material constants, κ = 2(*a*_10_ + *a*_01_)/(1−2υ) is the bulk modulus, and υ is the Poisson’s ratio. The parameters used (Table 1) ensure sufficient stiffness for the aponeuroses for a representative role in force transmission and providing muscular integrity as in real muscle.

### LFMM Model Features and Definitions

**FIGURE 2:**
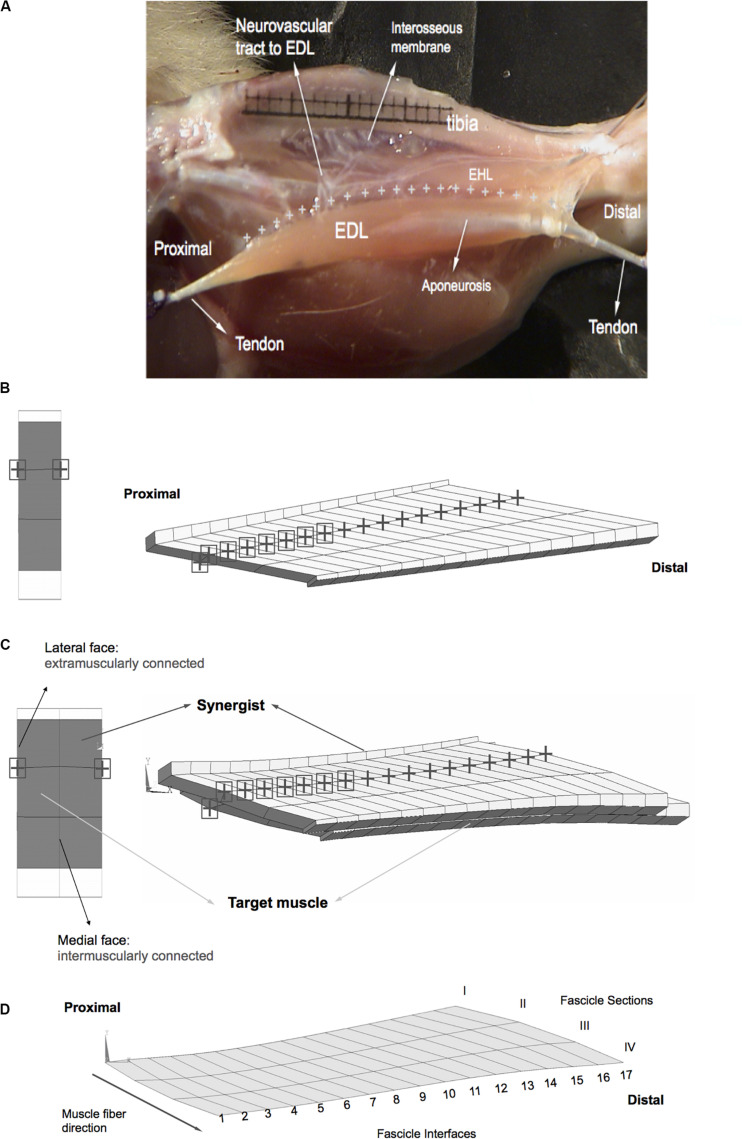
Anatomy and geometry of the model cases studied. **(A)** An image adapted from a previous experimental and anatomical study (Yucesoy et al., 2003b). The EDL is shown after removal of the tibialis anterior muscle of the anterior crural compartment, whereas the other muscle, EHL is not removed in this image. Extramuscular connections connect the EDL all along the muscle (marked by “+” signs) to the tibia, part of interosseal membrane and anterior intermuscular septum. This structure includes the neurovascular tract (i.e., the connective tissue structure containing nerves and blood vessels) and comprises a pathway of extramuscular myofascial force transmission. The geometry of modeled muscles is defined by the contour of a longitudinal slice of the rat EDL muscle belly **(B,C)**. Three muscle elements in-series define a large fascicle and sixteen fascicles in-parallel make up the muscle. Fascicles terminate in rigidly linked aponeurosis elements, which increase in thickness toward tendon ends. **(B)** Extramuscularly connected muscle. The nodes of the matrix mesh marked by a black “+” sign have extramuscular connections to mechanical ground and the nodes marked also by a black square have stiffer connections. A proximal view in the undeformed state is shown on the left-hand side. Locations of the extramuscular connections are indicated using the same symbols as in the view of the longitudinal plane. **(C)** Epimuscularly connected muscle. The target muscle is intermuscularly connected to the synergist muscle: the corresponding nodes of the matrix meshes of the two models are linked elastically along their medial faces (shown by black circles on the left-hand side). The synergist is kept at a fixed length, whereas the target muscle is lengthened distally. Both of the models are also connected extramuscularly: the nodes of the matrix mesh marked by a black “+” sign have connections to mechanical ground. The nodes marked also by a black square have stiffer extramuscular connections. A proximal view of the models in the undeformed state is shown on the left-hand side. Also in this view, the location extramuscular connections of the model are marked. **(D)** Fascicle sections I–IV and fascicle interfaces 1–17 are associated with model geometry.

Muscle length is defined as the distance between the proximal and distal muscle ends (Figure 2). *Initial muscle length* equals 28.7 mm. Three muscle elements in-series define a large fascicle (bundle of muscle fibers) and 16 fascicles in-parallel make up the muscle. An *isometric* condition describes an analysis in which the proximal and distal muscle ends are fixed; hence, the muscle length is globally kept constant. However, muscle fascicles run between the proximal and distal aponeuroses and can locally deform depending on the mechanical conditions imposed. It is assumed that, at the initial muscle length in the passive state, the sarcomeres arranged in series within muscle fibers have identical lengths. Local strain, as a measure of change of length, reflects the lengthening (positive strain) or shortening (negative strain) of sarcomeres. Note that zero strain in the model represents the undeformed state of sarcomeres (i.e., sarcomere length ≅ 2.5 μm) in the passive condition at initial muscle length. Fiber direction strain within the fiber mesh of the LFMM model was used to assess the non-uniformity of lengths of sarcomeres arranged in-series within muscle fibers (referred to as serial distribution).

### Muscle Models Studied

Extensor digitorum longus (EDL) muscle of the rat was modeled. This muscle is a unipennate muscle with a minimal variation of muscle fiber direction within the muscle belly. The muscle’s relatively simple geometry is suitable for the present modeling focused on assessment of effects of epimuscular myofascial loads on local muscle strains. The geometry of the model was defined by the contour of a longitudinal slice at the mid-muscle belly. Three models were studied:

(1) *Isolated muscle*: This muscle was kept fully isolated from its surroundings.
(2) *Extramuscularly connected muscle*: In order to model the muscles’ extramuscular connections (Figure 2A) and to account for their continuity with the muscular ECM, a set of nodes of the matrix mesh were linked using spring elements (COMBIN39, from the element library of ANSYS 12.0) to a set of fixed points (Figure 2B). In a previous experimental and anatomical study, the location of the extramuscular connections of the EDL muscle were determined to be at one-third of the fascicle length from the most proximal side of each muscle fascicle (Yucesoy et al., 2003b). In the muscle mechanics part of that study, those connections were dissected to a maximum possible extent, without endangering circulation and innervation and the part supporting the neurovascular tract to the EDL were shown to be much stiffer than the rest of the connective tissues. Taking into account those previous findings, our modeling considerations were: (1) the set of fixed points comprising “mechanical ground” represent bone, which is assumed to be rigid. (2) The spring elements modeling the muscles’ extramuscular connections were set to be uniaxial and have linear length–force characteristics. (3) Initially (i.e., muscle length = 28.7 mm, and before changing any of the tendon positions), the fixed points and the corresponding nodes of the model were at identical locations (i.e., the spring elements modeling the muscles’ extramuscular connections were at a length of zero). (4) The higher stiffness of the connective tissues constituting the neurovascular tract is taken into account by making the seven most proximal links to the muscle stiffer than the distal ones. Stiffness values determined previously (Yucesoy et al., 2003b) were used for the more compliant linkages, whereas a higher stiffness is preferred for the proximal ones (i.e., *k* = 0.25 unit force/mm for stiffer proximal part and, *k* = 0.033 unit force/mm for the remaining links).
(3) *Epimuscularly connected muscle*: This model aims at representing the principles of a muscle in its *in vivo* context. Hence, it includes the muscle’s extramuscular connections together with intermuscular connections to neighboring muscle. In order to achieve that, two muscle models with geometries identical to that in (1) were intermuscularly connected: the corresponding nodes of the matrix meshes of the two models were linked elastically along their medial faces (Figure 2C). Extramuscular linkages remained in the lateral faces of each model. For both inter- and extramuscular links, the spring element COMBIN39 was used with linear stiffness characteristics. A suitable stiffness value (*k* = 0.2 unit force/mm) was used for the intermuscular elements to provide sufficient mechanical interaction between modeled muscles.

### Cases Studied

**FIGURE 3:**
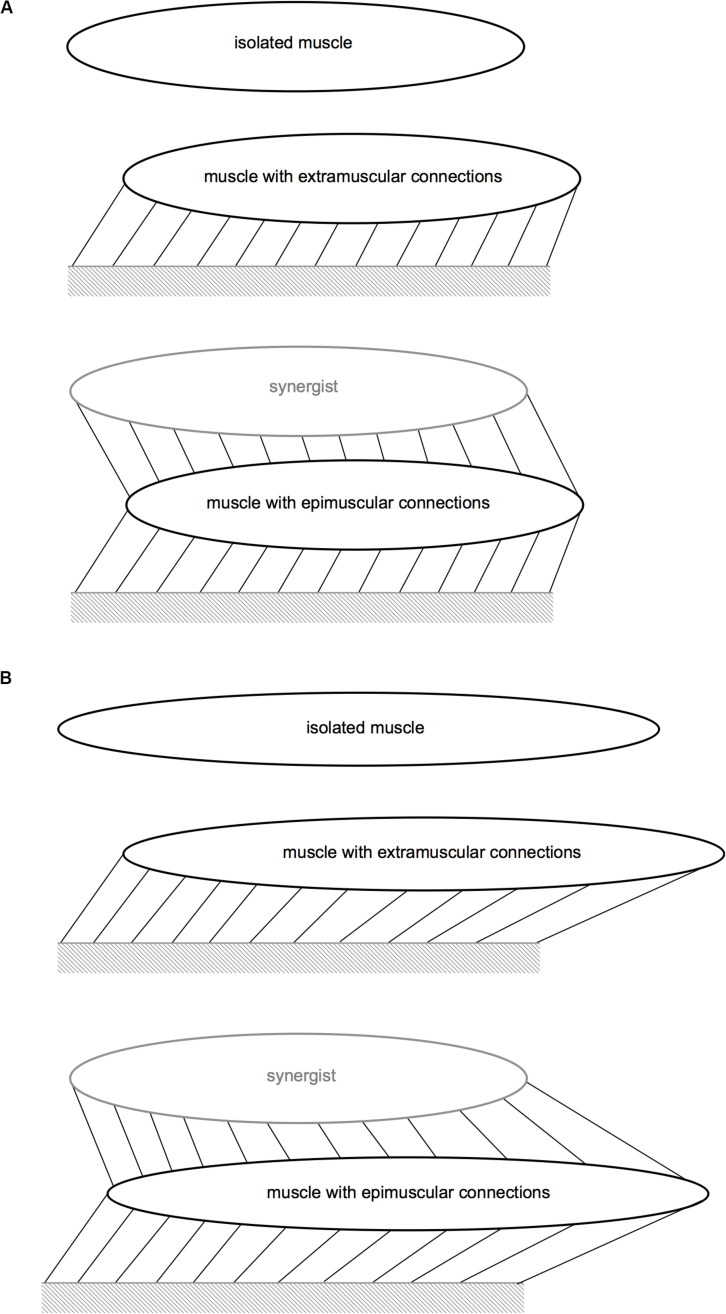
Schematic representation of the protocols modeled. **(A)** Protocols for Case I and Case III. **(B)** Protocol for Case II. The links that connect the muscle to the mechanical ground represent the extramuscular connective tissue, and those that connect the muscle to the synergistic muscle represent the intermuscular connections. Extra- and epimuscularly connected muscles illustrate stretching of these links with imposed muscle relative position change (Cases I–III) and length imposed (Case II).

In order to assess the effects of extra- and epimuscular myofascial loads on local length changes along the muscle fiber direction, three different cases were studied (Figure 3):

#### Case I: Passive Isometric Muscle, With Imposed Relative Position Changes

All three muscle models were studied in the passive state. The target muscles’ length was kept constant at the initial muscle length. However, relative position changes were imposed for extra- and epimuscularly connected ones in order to assess the effects of myofascial loads developed on muscle fiber direction strains. Implementation: the proximal and distal ends of the target muscle were displaced distally by 2 mm.

#### Case II: Passive Lengthened Muscle

All three muscle models were studied in the passive state. The target muscles were further lengthened distally, which for the extra- and epimuscularly connected ones also elevates myofascial loads via more pronounced muscle relative position changes. Implementation: Subsequent to the relative position change imposed as described above, the target muscle’s distal end was displaced distally by 2 mm.

#### Case III: Active Isometric Muscle, With Imposed Relative Position Changes

All three muscle models were fully activated, and their lengths were kept constant at the initial muscle length. However, relative position changes were imposed for the extramuscularly connected muscle and the epimuscularly connected target muscle, in order to assess the effects of myofascial loads developed on muscle fiber direction strains. Implementation: the proximal and distal ends of the target muscle were displaced distally by 2 mm.

Note that Case I actually does not even involve any muscle lengthening, but aims at showing that solely relative position changes may lead to occurrence in different parts of passive muscle with shortening as well as lengthening in muscle fiber direction due to myofascial loads. However, Case II does involve also muscle lengthening, which case directly addresses plausibility of shortened parts occurring within a passively lengthened muscle due to myofascial loads. Finally, Case III aims at plausibility of lengthened parts occurring within an activated isometric muscle. Note that this case represents a strict scenario for the specific aim as activated isolated muscle at initial length should involve exclusively shortening.

### Solution Procedure

The analysis type used in ANSYS was static and large strain effects were included. During the entire solution procedure, the models studied were stable and no mesh refinement was performed. A force-based convergence criterion was used with a tolerance of 0.5%.

At the passive state, the activation coefficient b_3_ (Eq. 6) equaled 0 (for Cases I and II). For Case III, maximal activation of the muscles modeled was achieved by increasing b_3_ incrementally up to 1, using fixed increments.

### Processing of Data

Local fiber direction strain indicated lengthening and shortening of sarcomeres, with zero strain representing the undeformed state of the sarcomeres. Strain (ε_22_) along nodes of serial fascicle interfaces (I–IV) were studied across fascicle interfaces 1–17 to quantify deformation patterns throughout muscle in isolated muscle, extramuscularly connected muscle, and epimuscularly connected muscle. For the epimuscularly connected muscle, the lateral, i.e., the extramuscularly connected face, and the medial, i.e., the inter-muscularly connected face, were assessed separately.

Epimuscular myofascial loads were calculated as the fiber direction components of nodal reaction forces on the epimuscular linking elements, and assessed as normalized with respect to the largest epimuscular myofascial load value observed in the three cases studied. The epimuscular myofascial loads, which cause the most pronounced muscle fiber direction length changes, were presented.

## Results

### Case I: Passive Isometric Muscle, With Imposed Relative Position Changes

**FIGURE 4:**
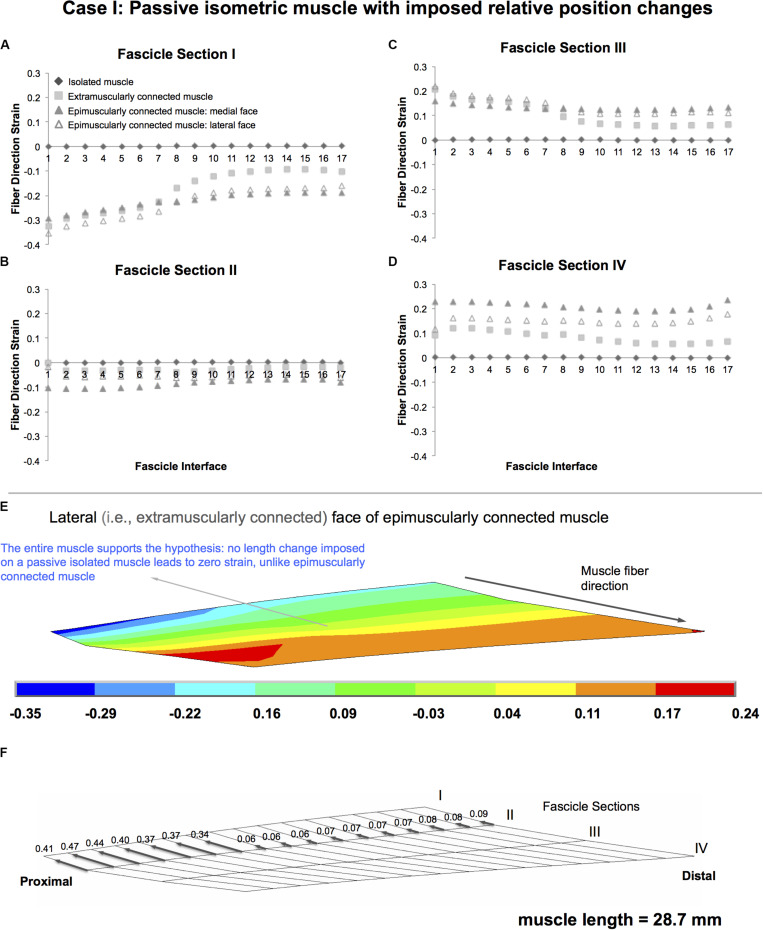
Results presented for Case I: Passive isometric muscle, with imposed relative position changes. **(A–D)** Fiber direction strains after relative position change for passive target muscle at 28.7 mm length are plotted per fascicle sections I–IV for each fascicle interface 1–17 in isolated muscle, in extramuscularly connected muscle, in medial and lateral faces of epimuscularly connected muscle. **(E)** Color strain contour plots exemplified for epimuscularly connected muscle model’s lateral face. The muscle parts that support the hypothesis are marked. **(F)** Myofascial loads due to extramuscular connections are calculated in local fiber direction, normalized with respect to the largest epimuscular myofascial load values observed in the three cases studied, and are depicted proportionately with glyphs on the lateral face of the target muscle.

Isolated isometric muscle shows the pre-defined zero strain condition as a reference for muscle with no myofascial loads acting on (Figure 4). In contrast, both extramuscularly and epimuscularly connected muscles show both shortening (Figures 4A,B—fascicle sections: I and II, maximally 35.3% in the lateral face of epimuscularly connected muscle), and lengthening (Figures 4C,D—fascicle sections: III and IV, maximally 23.6% in the medial face of epimuscularly connected muscle) in muscle fiber direction. For the lateral face of the epimuscularly connected muscle, which shows the most pronounced strain effects (Figure 4E), the myofascial loads (originating from extramuscular linking elements) are proximally directed owing to distally imposed muscle position changes. These loads, normalized with respect to the maximum epimuscular load calculated for Case II, range between 0.34 and 0.41 for the proximal nodes with stiffer extramuscular connections, and are below 0.09 for the remainder less stiff connections (Figure 4F). Despite the fact that no length changes were imposed globally on the muscle, these loads lead to shortening of the proximal part of the muscle (i.e., fascicle sections I and II). In return, the distal parts of the muscle (i.e., fascicle sections III and IV) show lengthening.

### Case II: Passive Lengthened Muscle

**FIGURE 5:**
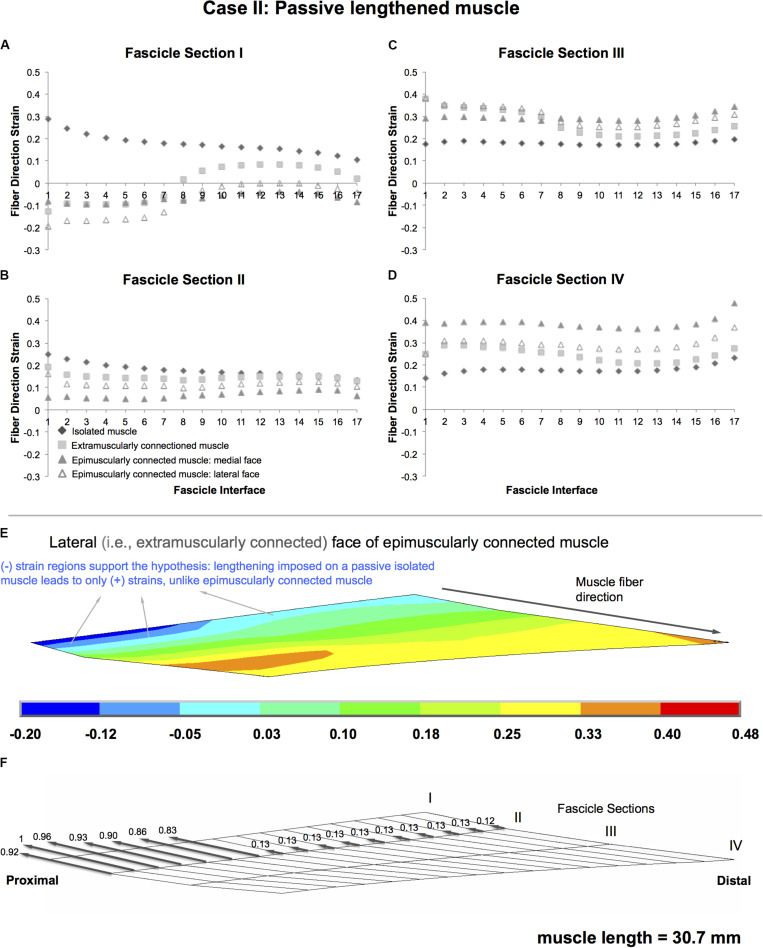
Results presented for Case II: Passive lengthened muscle. **(A–D)** Fiber direction strains after relative position change for passive target muscle at 30.7 mm length are plotted per fascicle sections I–IV over fascicle interfaces 1–17 for isolated muscle, muscle with extra-muscular connections alone, and for medial and lateral faces of muscle with inter- and extra-muscular connections. **(E)** Color strain contour plots exemplified for epimuscularly connected muscle model’s lateral face. The muscle parts that support the hypothesis are marked. **(F)** Myofascial loads due to extramuscular connections are calculated in local fiber direction, normalized with respect to the largest epimuscular myofascial load values observed in the three cases studied, and are depicted proportionately with glyphs on the lateral face of the target muscle.

Isolated lengthened muscle shows lengthening in all fascicle interfaces, for all fascicle sections in muscle fiber direction (maximally by 28.7, 24.9, 19.6, and 23.0% in fascicle sections I–IV, respectively) (Figure 5). However, the extramuscularly connected muscle involves muscle fiber direction lengthening in most parts of the muscle but also shortening in some regions (fascicle section I in fascicle interfaces 1-7, up to 12.7%). This shortening effect is more pronounced in the epimuscularly connected muscle where shortening in the muscle fiber direction is shown in all fascicle interfaces of fascicle section I (maximally by 19.5% in the lateral face, Figure 5E). Yet, presence of muscle fiber direction shortening is not the only difference. Compared to the isolated muscle, note also the different amplitudes of muscle fiber direction length changes shown in other parts: the extramuscularly connected muscle shows less pronounced muscle fiber direction lengthening in fascicle section I and II (maximally 19.2%, Figure 5B) and more pronounced muscle fiber direction lengthening in fascicle section III and IV (maximally 38.0%, Figure 5C). The epimuscularly connected muscle shows even lesser pronounced muscle fiber direction lengthening in fascicle section II (maximally 16.1%, Figure 5B) and even more pronounced muscle fiber direction lengthening in fascicle section III and IV (maximally 47.7%, Figure 5D). These effects are ascribed to proximally directed epimuscular myofascial loads acting on these muscles (Figure 5F shows those of the lateral face of the epimuscularly connected muscle). These loads range between 0.83 and 1 on the proximal nodes with stiffer extramuscular connections, and equal 0.13 for the majority of the remainder less stiff connections. As the muscles in this case are lengthened, the epimuscular myofascial loads manipulate the imposed muscle fiber stretch leading to elevation of lengthening in fascicle sections III and IV. However, they limit (fascicle section II) and even beat lengthening in the proximal parts of the muscle causing shortening fascicle section I.

### Case III: Active Isometric Muscle, With Imposed Relative Position Changes

**FIGURE 6:**
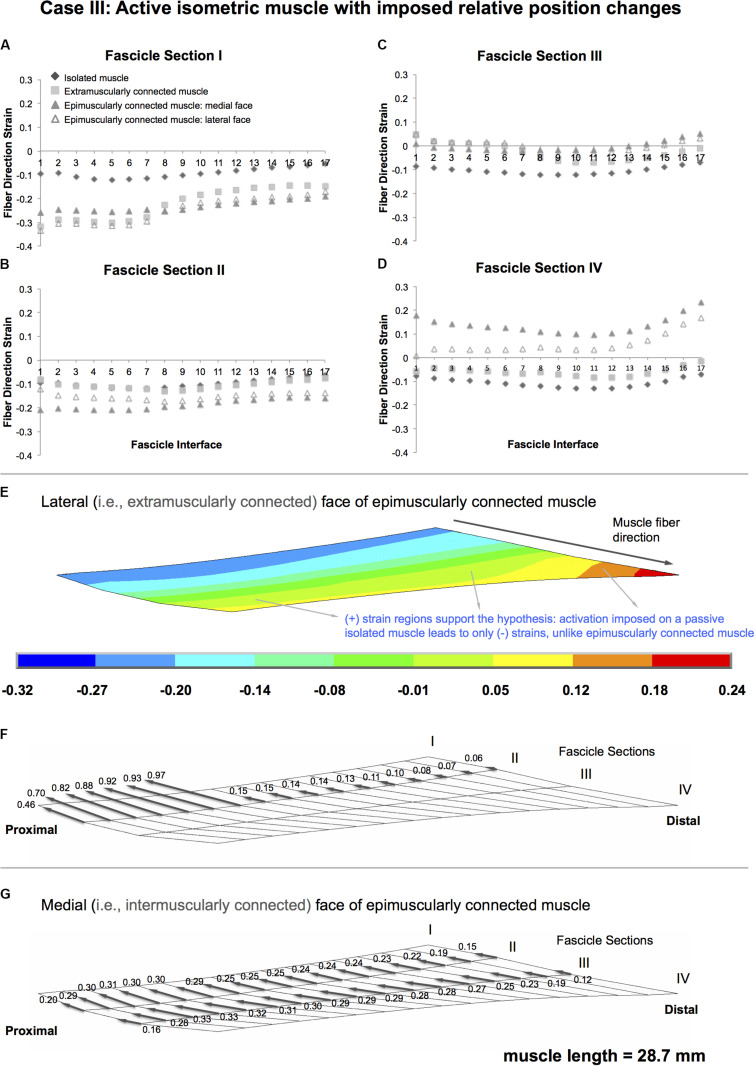
Results presented for Case III: Active isometric muscle, with imposed relative position changes **(A–D)** Fiber direction strains after relative position change for active target muscle at 28.7 mm length are plotted per fascicle sections I–IV for each fascicle interface 1–17 in isolated muscle, muscle with extra-muscular connections alone, and medial and lateral faces of muscle with inter- and extra-muscular connections. **(E)** Color strain contour plots exemplified for epimuscularly connected muscle model’s lateral face. The muscle parts that support the hypothesis are marked. **(F)** Myofascial loads due to extramuscular connections are calculated in local fiber direction, normalized with respect to the largest epimuscular myofascial load values observed in the three cases studied, and are depicted proportionately with glyphs on the lateral face of the target muscle. **(G)** Similarly, myofascial loads due to intermuscular connections are calculated and depicted proportionately with glyphs on the medial face of the target muscle.

Isolated muscle shows only shortening regions upon isometric contraction, with an average strain of 10.0% (maximally by 12.2, 11.9, 12.2, and 13.0% in fascicle sections I–IV, respectively) (Figure 6). In contrast, extra- and epimuscularly connected muscles show a more complex muscle fiber direction strain pattern including not only shortening, but also lengthening: (1) for fascicle sections I and II (Figures 6A,B), both muscles show a more pronounced shortening compared to the isolated muscle (up to 33.3%). (2) On the other hand, for fascicle section III (Figure 6C), both muscles show also certain lengthening in some fascicle interfaces (1–6 and 14–17, up to 5%). (3) Notably, for fascicle section IV (Figure 6D), the epimuscularly connected muscle shows lengthening in both lateral (up to 16.8%) and medial (up to 23.4%, Figure 6E) faces. These more complex muscle fiber direction strain patterns are ascribed to proximally directed epimuscular myofascial loads acting on these muscles. Figure 6F shows those on the lateral face (ranging between 0.46 and 0.97 for the proximal nodes with stiffer extramuscular connections and below 0.15 elsewhere) and Figure 6G shows those on the medial face (ranging between 0.33 and 0.12) of the epimuscularly connected muscle. As the muscles in this case are activated, the epimuscular myofascial loads manipulate the contraction-induced muscle fiber shortening. This leads to elevation of shortening in fascicle sections I and II as epimuscular myofascial loads shorten the proximal muscle elements. However, they limit and even overcome shortening as they stretch the distal muscle elements. This causes lengthening in fascicle sections III and IV.

Note that, further analyses (please see Supplementary Material) studying identical cases as above, but with higher stiffness inter- and extramuscular connections yield more emphasized shortening in passively lengthened muscle and more emphasized lengthening in active isometric muscle, and the opposite for lower stiffness inter- and extramuscular connections. Those findings together with those of additional analyses studying effects of reduced imposed muscle length in Case II sustain the role of myofascial loads in manipulating local length changes along muscle fibers.

## Discussion

The present modeling results indicate that muscle fiber direction strains in a truly isolated muscle and those in muscle in its *in vivo* context of connective tissue integrity would be very different. Note that, also for the truly isolated muscle, the findings show that muscle fibers operating within a whole muscle will not show a uniform muscle fiber direction strain distribution. This is because when the muscle’s geometry and the boundary conditions are taken into account the mechanical equilibrium dictates length variations. For example, for an isometric muscle, muscle activation will not be able to cause shortening in the restrained muscle ends, hence the muscle fibers located proximally and distally shorten less than the ones located in the middle parts of the muscle belly. Moreover, if the muscle geometry is asymmetrical, the mechanical equilibrium dictates non-uniformity of the muscle fiber direction strains along the length of the muscle fibers. The presently modeled EDL muscle is asymmetrical and hence shows such length change variability. However, a truly isolated muscle fiber studied outside the muscle belly may show uniform strains along its length. This is bound to certain assumptions though. The muscle fiber’s ends need to be fixed perfectly straight, so that no stress concentration occurs, which may, based on Saint Venant’s principle in mechanics (Toupin, 1965) alter the length changes at least within a part of the muscle fiber as long as its diameter. Also the mechanical properties of the muscle fiber need to be identical along its entire length. Nevertheless, the truly isolated muscle models studied presently did show one thing in common. That is, despite their non-uniformity, the muscle fiber direction strains were of the same direction, i.e., zero for Case I, all positive indicating lengthening for Case II, which involves a lengthened passive muscle and all negative indicating shortening for Case III, which involves an isometric activated muscle. In contrast, the extra and epimuscularly connected muscles showed non-uniform muscle fiber direction strains, which typically involved also different strain directions as well. Findings, which may appear unexpected, are shown particularly in Case II featuring shortened parts in a lengthened muscle despite being passive and in Case III featuring lengthened parts in a muscle, which is only activated without length changes imposed.

In addition to pioneering studies showing heterogeneous sarcomere lengths in human muscle *ex vivo* (van Eijden and Raadsheer, 1992) and in animal muscle *in situ* (Willems and Huijing, 1994), evidence on inhomogeneous length distribution of sarcomeres has accumulated for animal (Moo et al., 2016) and human (Lichtwark et al., 2018) tibialis anterior muscle *in vivo* at multiple sites using second harmonic generation imaging. Therefore, sarcomere length measurements from a small region may not be representative of the entire muscle. A recent study in which tissue loading was imposed by joint angle changes has shown inhomogeneity of along muscle fiber strain distributions in human muscle upon passive extension of the knee (Pamuk et al., 2016): MRI-based deformation analyses to determine local strains and DTI-based tractography to determine muscle fiber direction combined, the study showed that passively imposed proximal lengthening of human medial gastrocnemius (GM) yields both lengthened and shortened sections along the same fascicles. Using the same techniques, studying sustained submaximal isometric plantar flexion effort showed non-uniform strain distributions along muscle fascicles to include not only shortening but also lengthening (Karakuzu et al., 2017). Those effects were explained by EMFT in both studies: as locally epimuscular myofascial loads can affect the mechanical equilibrium, shortened muscle fascicle parts in a muscle stretch scenario and lengthened muscle fascicle parts in a muscle activation scenario was considered conceivable. This was supported by an assessment of local first principal strains within the segmented and visualized neurovascular tracts. Stretch on those collagen reinforced structures indicated exposure to epimuscular myofascial loads (Karakuzu et al., 2017). The locations within the GM where the neurovascular tracts were entering into the muscle were those that showed elevated muscle fiber direction length changes exemplifying the role of intermuscular mechanical interactions. Those strains were interpreted as sarcomere length changes along the GM fascicles *in vivo*. However, it must be noted that the resolution in these studies are in the order of millimeters, which is much coarser than the length of a sarcomere, and corresponds to sections of fascicle bundles. Also, the measurements involve strain, not length. Thus, strain non-uniformity found does not directly correspond to varying sarcomere lengths. Yet, these techniques are up-to-date assets: while DTI-based tractography alone is a powerful tool providing repeatable (Heemskerk et al., 2010) anatomical information about human muscle fibers *in vivo* for both upper (Froeling et al., 2012) and lower extremity (Sinha et al., 2011), coupling it with sequences providing tissue deformation or deformation rate such as cine phase contrast in order to achieve fiber direction information has been limited to few slices at a time (e.g., Felton et al., 2008). Time and subject’s effort demands and more importantly dynamic nature of cine phase contrast-imaging experiments cause that. This technique yields tissue strains more directly (Asakawa et al., 2003) than MRI image registration techniques, but the resolution is low and an assumption is made that the static DTI acquisition represents the tissue properties at a selected joint angle during dynamic joint motion.

Techniques combining MRI and DTI analyses allow studying effects of intermuscular mechanical interactions in human muscles *in vivo* but as in other MRI modalities, provide kinematic information only. Although plantar-flexion force measured within the MRI scanner fed visually to the subject during acquisition (Karakuzu et al., 2017) helped standardizing the subjects’ sustained submaximal activation, the muscle force remains unknown. Therefore, in MRI analyses, an explanation of the findings based directly on force equilibrium of the muscle is not possible. However, finite element modeling allows addressing mechanical principles of that, and the present findings show the role of epimuscular myofascial loads on along muscle fiber strain distributions. It was pointed out recently (Maas, 2019) that such modeling may be used to elaborate on the findings of Pamuk et al. (2016). The present study accomplishes that by three model cases, which exemplify scenarios that yield uniform direction of strains if EMFT mechanism is ignored, and strains of opposite directions and heterogeneous amplitudes occurring along the same fascicles if it is not.

Note that human muscle studied in MRI analyses and the rat EDL model studied presently is not equivalent, but structural and muscle relative position differences exist. Regarding structure: the unipennate longitudinal EDL slice modeled bares important simplifications compared to complex geometries and properties of human muscles. The epimuscularly connected model involves two identical muscles with separate tendons, which is also a major structural simplification. Regarding muscle relative position changes: According to measurements in cadavers, 90° knee angle change results in 8% length change in GM (Huijing et al., 2011), with 30° knee extension imposed in MRI analyses (Pamuk et al., 2016) plausibly corresponding to 3–4% change in muscle length. Although presently modeled muscle lengthening approximates 7%, in the human study, the ankle kept at 90° also imposes a stretch on the GM suggesting that a comparable muscle lengthening is conceivable. However, intermuscular mechanical interactions must differ. Present epimuscularly connected model limits the mechanical interaction of the target muscle to that with an adjacent muscle only. Modeled muscles’ interconnected faces provide direct, and neurovascular tracts connected at the mechanical ground provide indirect intermuscular interaction. In human, the target muscle is a part of the triceps surae with combined bipennate geometry and distally the muscles insert to the Achilles tendon, which also allows mechanical interaction (Tian et al., 2012). Moreover, the intact limb provides mechanical interaction for the gastrocnemius muscles also with antagonistic muscles. Due to the preserved ankle position, proximally imposed lengthening changes of their position relative to the remainder lower leg muscles. Therefore, more pronounced intermuscular relative position changes and stretch on interconnecting connective tissue structures is plausible. Note that location and stiffness of such connections are conceivably different not only across muscles and species, but also among individuals. Therefore, the amplitudes and directions of myofascial loads differ in both studies. Please see Supplementary Material for model elaboration on extra- and epimuscular connection stiffness’s and muscle relative position changes, which confirm the role of myofascial loads in the mechanism of along muscle fiber direction strain heterogeneity.

The rat EDL has been studied extensively experimentally (Ettema and Huijing, 1989; Huijing et al., 1998, 1994; Jaspers et al., 2002; Huijing and Baan, 2003) and using matching finite element modeling (Maas et al., 2003; Yucesoy et al., 2003a, 2005, b). Therefore, a good understanding of its mechanics and anatomy forms sound basis for present modeling. Previous work on epimuscularly connected bi-articular rat EDL generated several new viewpoints suggesting implications of the present findings. Unequal proximal and distal forces is one characteristic finding (Huijing and Baan, 2001). The force difference at each muscle length represents the net of epimuscular myofascial loads acting on the muscle belly. Modeling their effects inside the muscle led to an understanding that muscle length is not the sole determinant of isometric muscle force production. Instead, muscle relative position is a co-determinant because it manipulates epimuscular myofascial loads, which in return affect sarcomere lengths (Maas et al., 2003, 2004; Yucesoy et al., 2003a, 2006; Bernabei et al., 2015). Muscle fiber–ECM mechanical interactions are the core of this mechanism, e.g., limiting sarcomere shortening in botulinum toxin type-A (BTX-A) treated muscle (Turkoglu et al., 2014), which explains muscle length-dependent force reductions (Yaraskavitch et al., 2008) and narrowing of muscle length range of force exertion (Ateş and Yucesoy, 2014). Elevated ECM stiffness (Yucesoy and Ateş, 2018) yields more pronounced BTX-A effects in modeled due course of treatment (Turkoglu and Yucesoy, 2016). Epimuscular myofascial loads studied presently are important because they interfere with muscle fiber–ECM mechanical interactions. This may make muscle’s length–force characteristics vary in different conditions. It was shown intraoperatively in patients with cerebral palsy that spastic muscles’ force amplitude can change significantly by co-activating other muscles (Kaya et al., 2018, 2019, 2020).

## Conclusion

In the present study, we developed finite element models and studied different cases to explore the principles of the mechanisms of non-uniform strain distributions along the muscle fiber direction with a particular emphasis on strains opposing the imposed effect as shown in previous human studies *in vivo*. Assessments of muscle fiber direction strains and forces exerted on the muscle by the epimuscular connections showed that such strain heterogeneities are ascribed to epimuscular myofascial loads determined by muscle relative position changes.

## Data Availability Statement

The datasets generated for this study are available on request to the corresponding author.

## Author Contributions

CY and UP contributed to conception and design of the study and wrote sections of the manuscript. AC, UP, and CY contributed to the development of models and acquisition of the data. AC contributed to data processing. UP wrote the first draft of the manuscript. All authors contributed to interpretation of data for the work, manuscript revision, and read and approved the submitted version.

## Conflict of Interest

The authors declare that the research was conducted in the absence of any commercial or financial relationships that could be construed as a potential conflict of interest.
