# Supplementary material for: Principles of the Mechanism for Epimuscular Myofascial Loads Leading to Non-uniform Strain Distributions Along Muscle Fiber Direction: Finite Element Modeling
Source: Front Physiol. 2020 Jul 3;11:789. doi: 10.3389/fphys.2020.00789 (PMC7351515; doi:10.3389/fphys.2020.00789)
Supplement: Supplementary file 1 [file Data_Sheet_1.DOCX]

## 1) Effects of Stiffer Extra- and Inter-muscular Linkages on Muscle Fiber Direction Strain Non-uniformities

Hypothesis

Elevated extra- and inter-muscular linkage stiffness values make strains opposing those that are determined by the globally imposed conditions more pronounced.

Methods

Using four times stiffer extra- and inter-muscular linkage stiffness values than those used in the main text, the same three models (i.e., *isolated muscle*, *extramuscularly connected muscle* and *epimuscularly connected muscle*) were studied in the same three cases (i.e., *passive isometric muscle, with imposed relative position changes*, *passive lengthened muscle* and *active isometric muscle, with imposed relative position changes*). Inter- and extramuscular myofascial loads are calculated in local fiber direction, normalized with respect to the largest load values observed in the three cases studied, and exemplified.


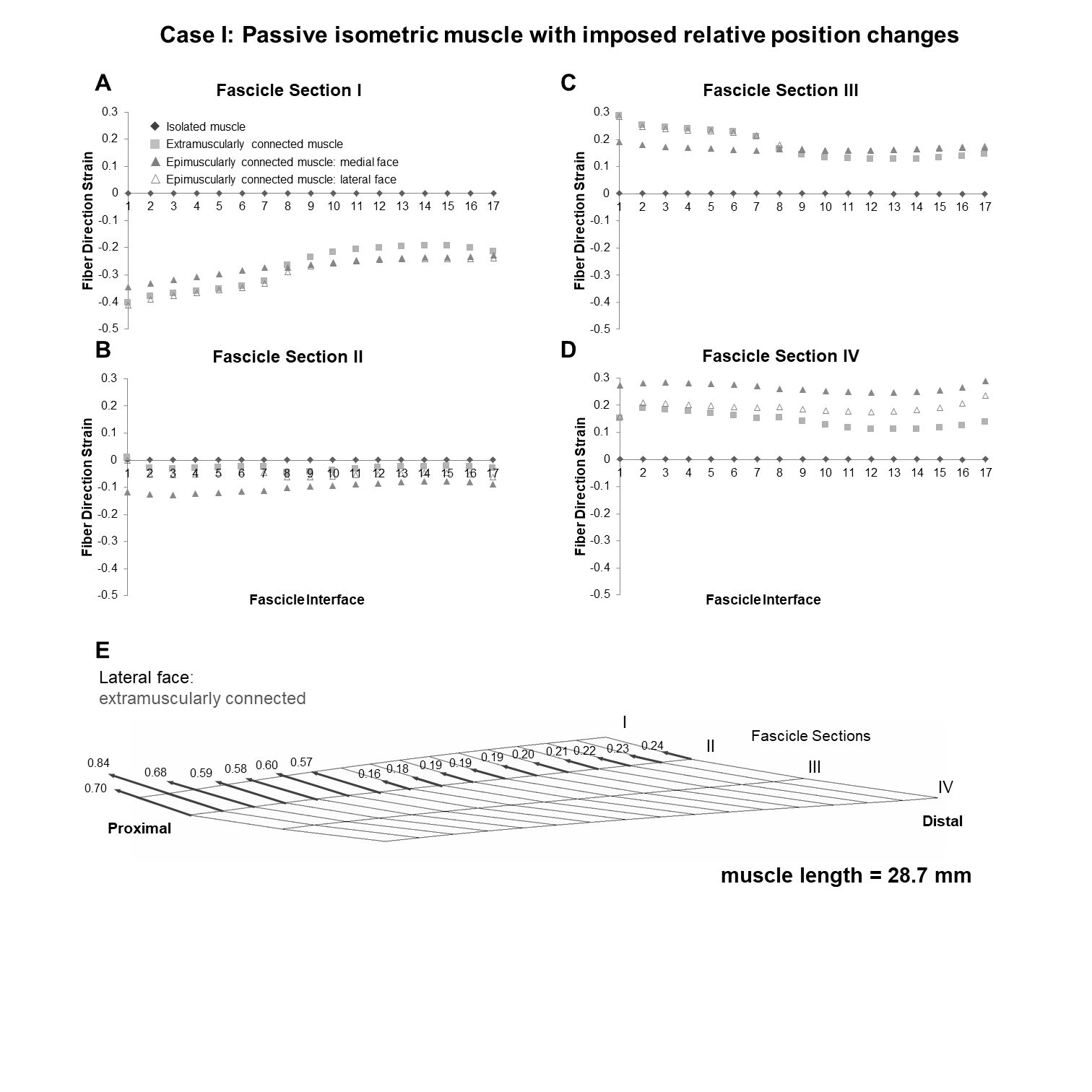


**Figure S1.** Fiber direction strains for passive target muscle at 28.7 mm length are plotted per fascicle sections I-IV **(A-D)** for each fascicle interface 1-17 in isolated muscle, extramuscularly connected muscle, and medial and lateral faces of epimuscularly connected muscle. **(E)** Extramuscular myofascial loads are depicted proportionately with glyphs on the lateral face of the target muscle.

Results

*Case I: Passive isometric muscle, with imposed relative position changes*

Isolated isometric muscle shows the pre-defined zero strain condition as a reference for muscle with no myofascial loads acting on. In contrast, both extramuscularly and epimuscularly connected muscles show both shortening (fascicle sections: I and II, maximally 40.5% (*vs. 35.3% in main text*) in the lateral face of epimuscularly connected muscle), and lengthening (fascicle sections: III and IV, maximally 29.0% (*vs. 23.6%*) in the medial face of epimuscularly connected muscle) in muscle fiber direction (Fig. S1 A-D).

Normalized epimuscular loads on the lateral face of epimuscularly connected muscle range between 0.57 and 0.84 for the proximal linkages, and are above 0.16 for remainder linkages (Fig. S1 E). Compared to the results shown in the main text, elevated extra- and inter-muscular linkage stiffness values yield in concert with the greater strain amplitudes also higher amplitudes of epimuscular loads.

**
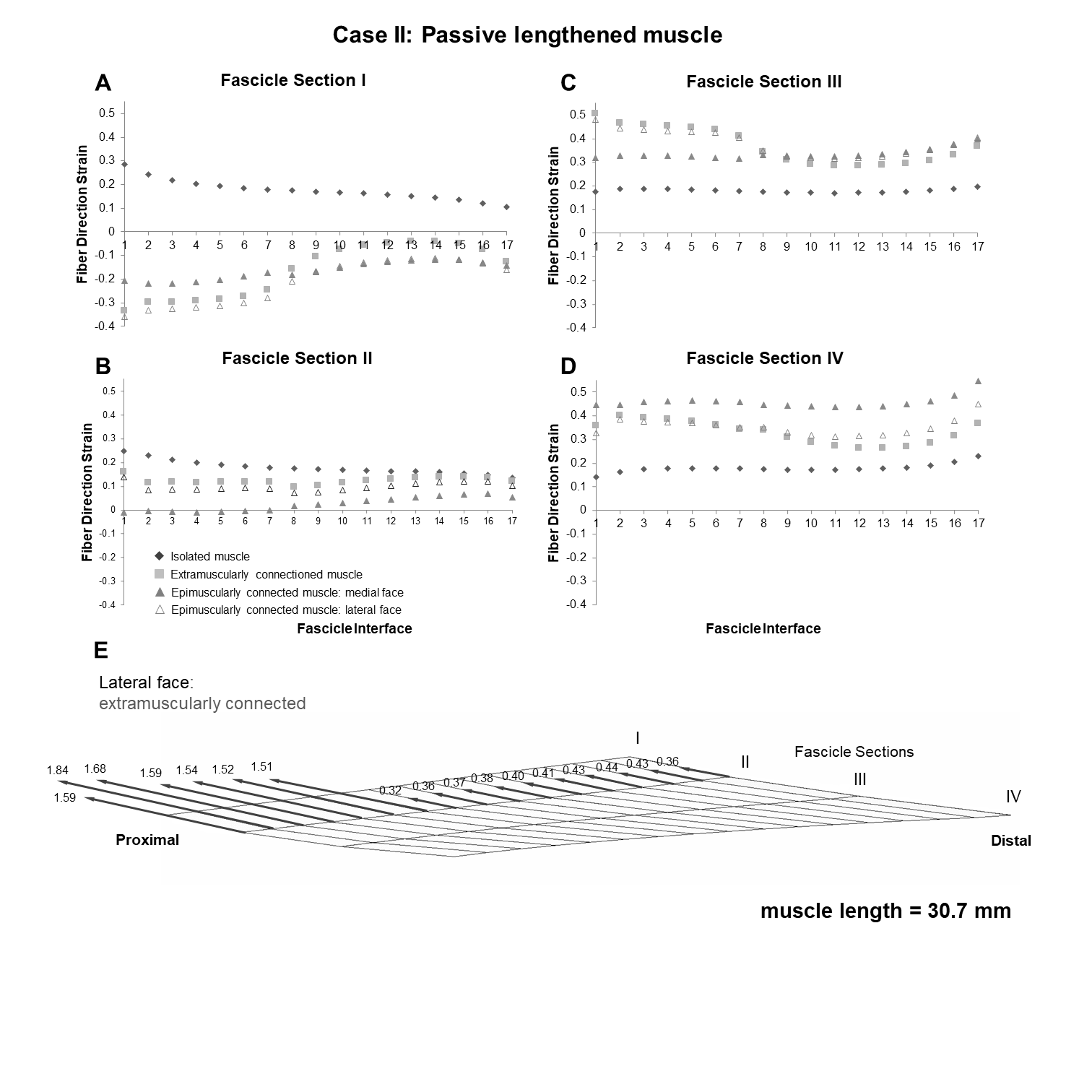
**

**Figure S2.** Fiber direction strains for passive target muscle at 30.7 mm length are plotted per fascicle sections I-IV **(A-D)** over fascicle interfaces 1-17 for isolated muscle, muscle with extra-muscular connections alone, and for medial and lateral faces of muscle with inter- and extra-muscular connections. **(E)** Extramuscular myofascial loads are depicted proportionately with glyphs on the lateral face of the target muscle.

**
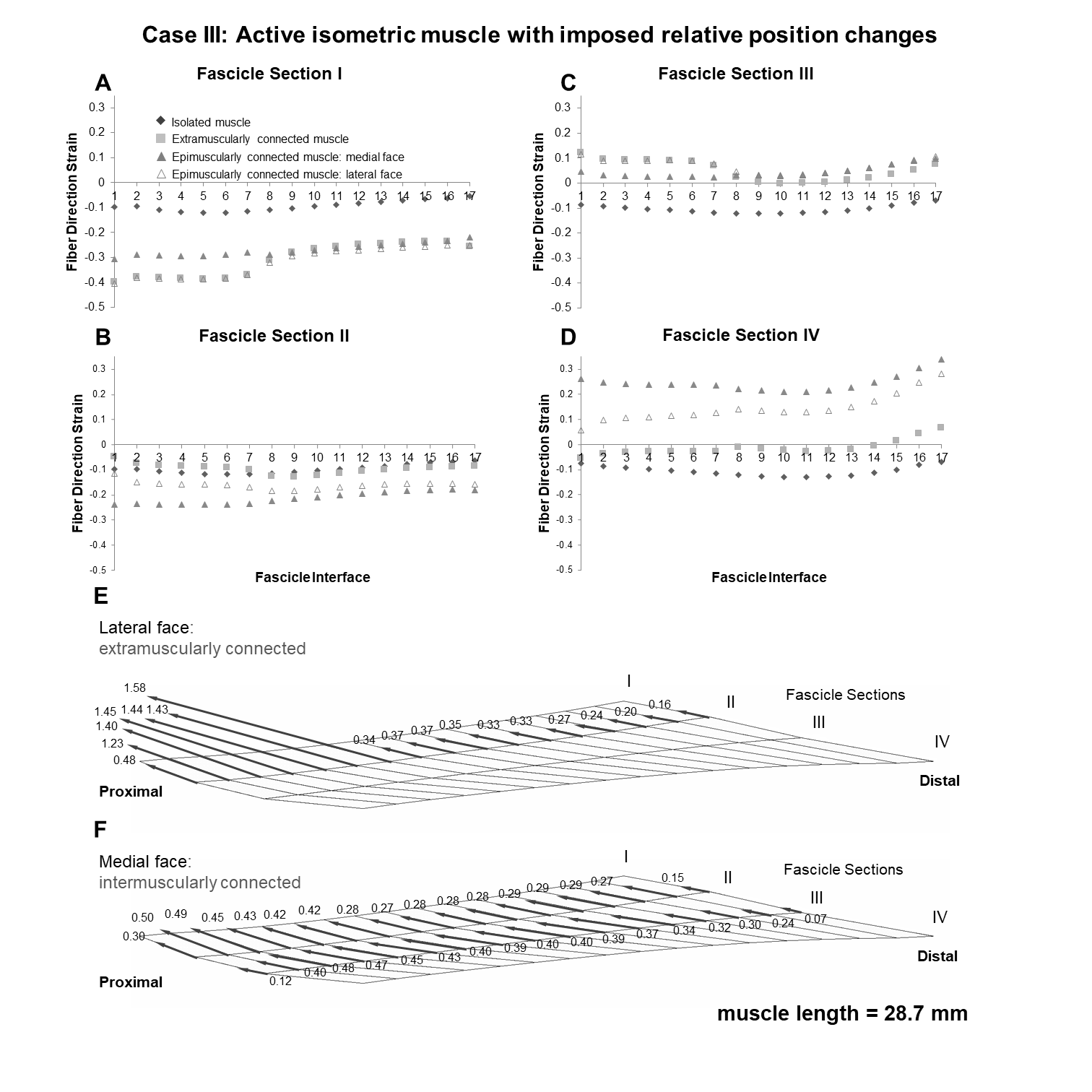
**

**Figure S3.** Fiber direction strains for active target muscle at 28.7 mm length are plotted per fascicle sections I-IV **(A-D)** for each fascicle interface 1-17 in isolated muscle, muscle with extra-muscular connections alone, and medial and lateral faces of muscle with inter- and extra-muscular connections. **(E-F)** extramuscular and intermuscular myofascial loads are depicted proportionately with glyphs on the lateral and medial face of the target muscle, respectively.

*Case II: Passive lengthened muscle*

Isolated lengthened muscle shows lengthening in all fascicle interfaces, for all fascicle sections in muscle fiber direction (maximally by 28.7, 24.9, 19.6 and 23.0% in fascicle sections I, II, III and IV, respectively). However, the extramuscularly connected muscle does show in addition to lengthening occurring in most parts of the muscle, also shortening in muscle fiber direction (fascicle section I in fascicle interfaces 1-7, up to 33.5% (*vs. 12.7% in main text*). This shortening effect is more pronounced in the epimuscularly connected muscle. Shortening in muscle fiber direction is shown in all fascicle interfaces of fascicle section I (maximally by 35.9% (*vs. 19.5%*) in the lateral face). Note that, myofascial loads acting on the extra- and epimuscularly connected muscles also limit (fascicle sections: I and II, maximally by 35.9% shortening and 15.9% lengthening, respectively) (Fig. S2 A-B) and elevate (fascicle sections: III and IV, maximally by 50.5 and 54.8%, respectively) lengthening in the remainder nodes (Fig. S2 C-D).

Normalized epimuscular loads on the lateral face of epimuscularly connected muscle range between 1.51 and 1.84 (*vs. 0.83 and 1 in main text*) for the stiffer proximal nodes, and are in excess of 0.32 (*vs. 0.13*) for remainder less stiff connections (Fig. S2 E). Compared to the results shown in the main text, elevated extra- and inter-muscular linkage stiffness values yield in concert with the more emphasized shortening in passive lengthened muscle, also higher amplitudes of epimuscular loads.

*Case III: Active isometric muscle, with imposed relative position changes*

Isolated muscle shows only shortening regions upon isometric contraction, with an average strain of -10.0% (maximally by 12.2, 11.9, 12.2 and 13.0% in fascicle sections I, II, III and IV, respectively) (Fig. S3). In contrast, extra- and epimuscularly connected muscles show both lengthening and shortening. Extramuscularly connected muscle involves lengthened regions in some fascicle interfaces (1-7 and 16-17) of fascicle section III, maximally up to 12.1% (*vs. 5% in main text*). Epimuscularly connected muscle on the other hand yielded lengthening in all nodes of fascicle section III, up to 11.5% maximally. This lengthening effect is more pronounced in epimuscularly connected muscle, especially in fascicle section IV which shows solely lengthened nodes (maximally up to 28.1% (*vs. 16.8%*) in its lateral face and up to 33.8% (*vs. 23.4%*) in its medial face). Note that myofascial loads on extra- and epimuscularly connected muscle results in even more emphasized shortening in fascicle section I, by 39.8 and 40.3%, respectively.

## Fig. S3E shows proximally directed epimuscular myofascial loads acting on lateral face of target muscle through extramuscular connections (ranging between 0.48 and 1.58 (*vs. 0.46 and 0.97 in main text*) for the stiffer proximal nodes and in excess of 0.16 for the remainder nodes) is increased and those acting through the intermuscular connections on the medial face now span a wider range of values (ranging between 0.50 (*vs. 0.33*) and 0.07), contributing to the increased emphasis on the complex strain pattern observed.

## 2) Effects of Less Stiff Extra- and Inter-muscular Linkages on Muscle Fiber Direction Strain Non-uniformities

Hypothesis

Reduced extra- and inter-muscular linkage stiffness values make strains opposing those that are determined by the globally imposed conditions less pronounced.

Methods

Halving the stiffness values of extra- and inter-muscular linkages compared to those used in the main text, the same three models (i.e., *isolated muscle*, *extramuscularly connected muscle* and *epimuscularly connected muscle*) were studied in the same three cases (i.e., *passive isometric muscle, with imposed relative position changes*, *passive lengthened muscle* and *active isometric muscle, with imposed relative position changes*). Inter- and extramuscular myofascial loads are calculated in local fiber direction, normalized with respect to the largest load values observed in the three cases studied, and a relevant one is exemplified.

Results

*Case I: Passive isometric muscle, with imposed relative position changes*

Isolated isometric muscle shows the pre-defined zero strain condition as a reference for muscle with no myofascial loads acting on. In contrast, both extramuscularly and epimuscularly connected muscles show both shortening (fascicle sections: I and II, maximally 30.0% (*vs. 35.3% in main text*) in the lateral face of epimuscularly connected muscle), and lengthening (fascicle sections: III and IV, maximally 19.8% (*vs. 23.6%*) in the medial face of epimuscularly connected muscle) in muscle fiber direction (Fig. S4 A-D).

Normalized epimuscular loads on the lateral face of epimuscularly connected muscle range between 0.24 and 0.30 for the proximal linkages, and above 0.04 for remainder linkages (Fig. S4 E). Compared to the results shown in the main text, reduced extra- and inter-muscular linkage stiffness values yield in concert with the smaller strain amplitudes also lower amplitudes of epimuscular loads.


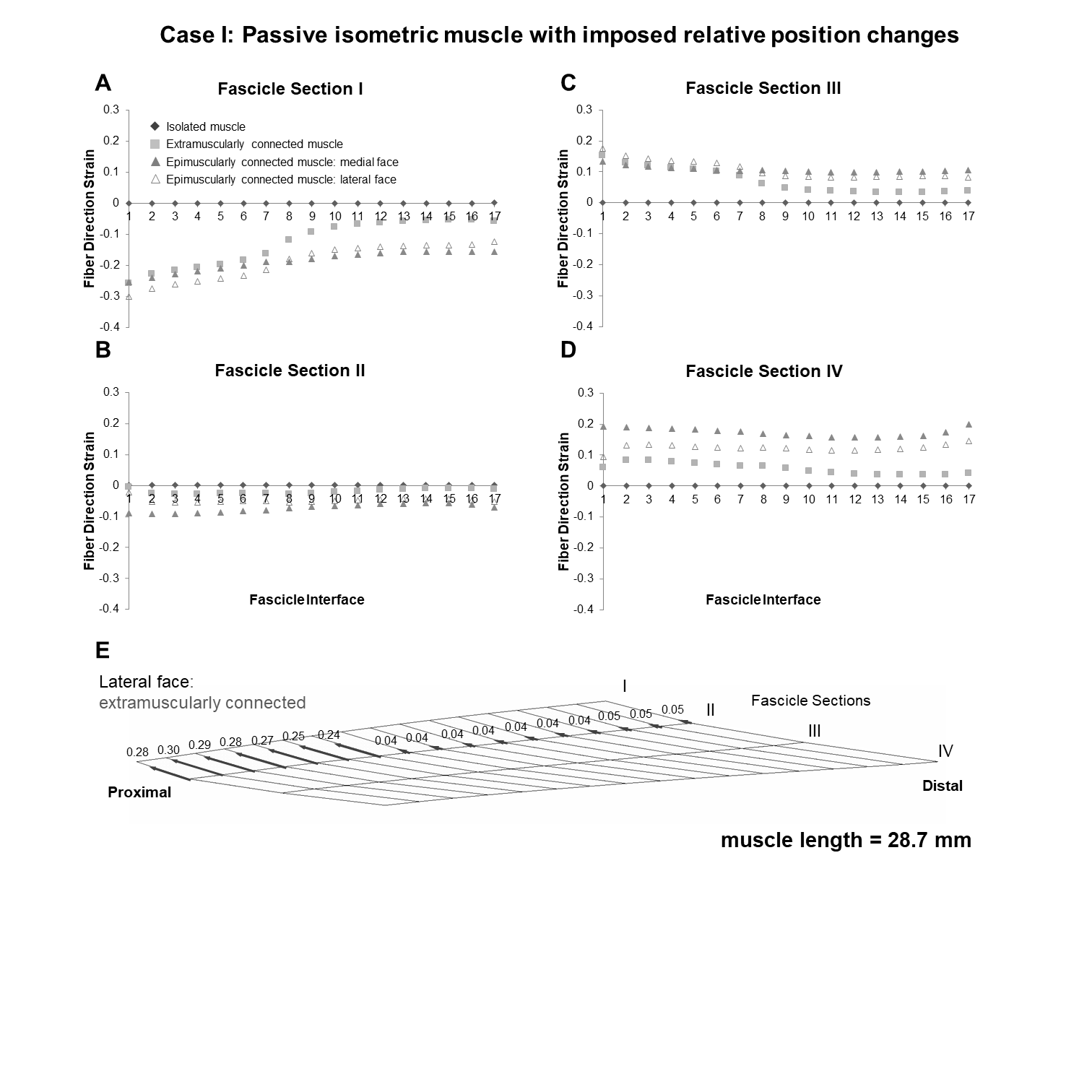


**Figure S4.** Fiber direction strains for passive target muscle at 28.7 mm length are plotted per fascicle sections I-IV **(A-D)** for each fascicle interface 1-17 in isolated muscle, extramuscularly connected muscle, and medial and lateral faces of epimuscularly connected muscle. **(E)** Extramuscular myofascial loads are depicted proportionately with glyphs on the lateral face of the target muscle.

**
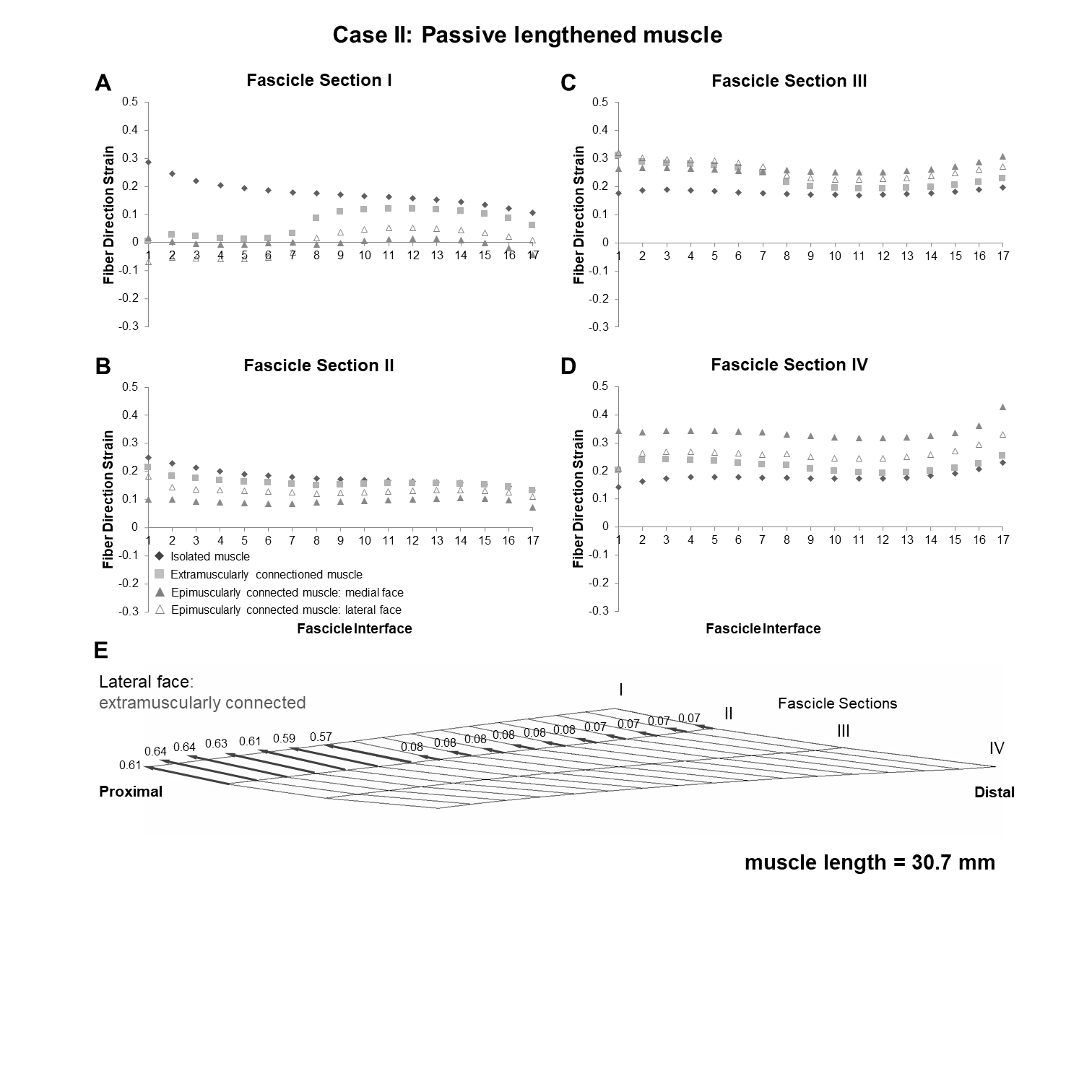
**

**Figure S5.** Fiber direction strains for passive target muscle at 30.7 mm length are plotted per fascicle sections I-IV **(A-D)** over fascicle interfaces 1-17 for isolated muscle, muscle with extra-muscular connections alone, and for medial and lateral faces of muscle with inter- and extra-muscular connections. **(E)** Extramuscular myofascial loads are depicted proportionately with glyphs on the lateral face of the target muscle.

*Case II: Passive lengthened muscle*

Isolated lengthened muscle shows lengthening in all fascicle interfaces, for all fascicle sections in muscle fiber direction (maximally by 28.7, 24.9, 19.6 and 23.0% in fascicle sections I, II, III and IV, respectively). Note that, extramuscularly connected muscle also shows lengthening in all fascicle interfaces, for all fascicle sections in muscle fiber direction (maximally by 12.0, 21.2, 31.1 and 25.3% in fascicle sections I, II, III and IV, respectively, *unlike 12.7% shortening shown in fascicle section I in main text*). However, the epimuscularly connected muscle does involve along lengthening occurring in most parts of the muscle, also shortening in muscle fiber direction (fascicle section I in fascicle interfaces 1-7, up to 6.9% (*vs. 19.5% in main text*) in its lateral face).

Normalized epimuscular loads on the lateral face of epimuscularly connected muscle range between 0.57 and 0.64 (*vs. 0.83 and 1 in main text*) for the stiffer proximal nodes, and are in excess of 0.07 (*vs. 0.13*) for remainder less stiff connections (Fig. S5 E). Compared to the results shown in the main text, reduced extra- and inter-muscular linkage stiffnesses yield in concert with the less emphasized shortening in passive lengthened muscle, also lower amplitudes of epimuscular loads.

**
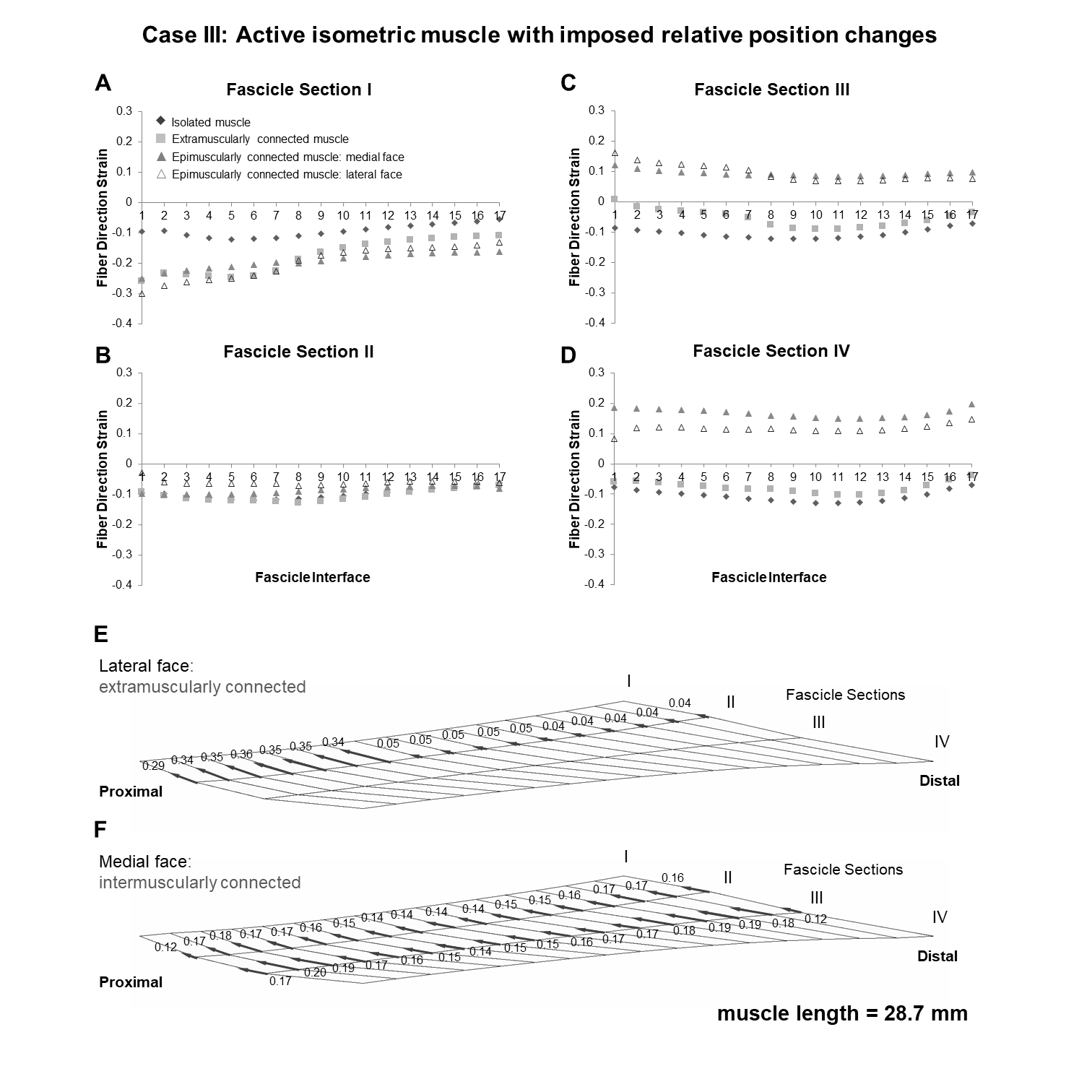
**

**Figure S6.** Fiber direction strains for active target muscle at 28.7 mm length are plotted per fascicle sections I-IV **(A-D)** for each fascicle interface 1-17 in isolated muscle, muscle with extra-muscular connections alone, and medial and lateral faces of muscle with inter- and extra-muscular connections. **(E-F)** extramuscular and intermuscular myofascial loads are depicted proportionately with glyphs on the lateral and medial face of the target muscle, respectively.

*Case III: Active isometric muscle, with imposed relative position changes*

Isolated muscle shows only shortening regions upon isometric contraction, with an average strain of -10.0% (maximally by 12.2, 11.9, 12.2 and 13.0% in fascicle sections I, II, III and IV, respectively) (Fig. S6). In contrast extra- and epimuscularly connected muscles show both lengthening and shortening. Extramuscularly connected muscle involves certain limited lengthening in regions in fascicle interface 1 of fascicle section III by 0.6% (*vs. maximally 5% in main text*). This lengthening effect is more pronounced in epimuscularly connected muscle, especially in fascicle section IV which shows solely lengthened nodes (maximally up to 16.3% (*vs. 16.8%*) in its lateral face and up to 19.8% (*vs. 23.4%*) in its medial face). Note that, myofascial loads on extra- and epimuscularly connected muscles lead also to less pronounced shortening in fascicle section I, by 26.1 and 30.1%, respectively.

Fig. S6E shows proximally directed epimuscular myofascial loads acting on lateral face of target muscle through extramuscular connections (ranging between 0.29 and 0.36 for the stiffer proximal nodes and in excess of 0.04 for the remainder nodes) is decreased and those acting through the intermuscular connections on the medial face now span a narrower range of values (ranging between 0.12 and 0.20).

## 3) Effects of Reduced Imposed Muscle Lengthening on Muscle Fiber Direction Strain Non-uniformities

Hypothesis

Reduced imposed muscle lengthening makes strains opposing those that are determined by the globally imposed condition less pronounced.

Methods

Using identical extra- and inter-muscular linkage stiffness values to those used in the modeling presented in main text, the same three models (i.e., *isolated muscle*, *extramuscularly connected muscle* and *epimuscularly connected muscle*) were studied in Case II (i.e., *passive lengthened muscle*). Two different amplitudes of reduced imposed muscle lengthening (i.e., 4% and 3% lengthening, corresponding to 29.8 mm and 29.5 mm muscle length, respectively) were studied compared to that imposed in the modeling presented in main text (i.e., 7% lengthening corresponding to 30.7 mm muscle length). Inter- and extramuscular myofascial loads are calculated in local fiber direction, normalized with respect to the largest load values observed, and exemplified.

Results

*Case II: Passive lengthened muscle (to muscle length 29.8 mm)*

Isolated lengthened muscle shows lengthening in all fascicle interfaces, for all fascicle sections in muscle fiber direction (maximally by 16.1, 13.7, 10.7 and 12.7% in fascicle sections I, II, III and IV, respectively). However, the extramuscularly connected muscle does show in addition to lengthening occurring in most parts of the muscle, also shortening in muscle fiber direction (fascicle section I, up to 23.3% (*vs. 12.7% in main text*). This shortening effect is more pronounced in the epimuscularly connected muscle. Shortening in muscle fiber direction is shown in all fascicle interfaces of fascicle section I (maximally by 28.0% (*vs. 19.5%*) in the lateral face). Note that, myofascial loads acting on the extra- and epimuscularly connected muscles also limit (fascicle sections: I and II, maximally by 28.0% shortening and 10.2% lengthening, respectively) (Fig. S7 A-B) and elevate (fascicle sections: III and IV, maximally by 31.8 and 37.9%, respectively) lengthening in the remainder nodes (Fig. S7 C-D).

Normalized epimuscular loads on the lateral face of epimuscularly connected muscle range between 0.57 and 0.71 (*vs. 0.83 and 1 in main text*) for the stiffer proximal nodes, and are in excess of 0.09 (*vs. 0.13*) for remainder less stiff connections (Fig. S7 E). Compared to the results shown in the main text, reduced imposed muscle lengthening yields lower amplitudes of epimuscular myofascial loads, because they are stretched less due to less pronounced relative position change of the target muscle. Yet, because also the imposed muscle length itself causes less pronounced lengthening in the muscle fiber direction, these loads lead to an even more pronounced local shortening effect compared to those shown in the main text.


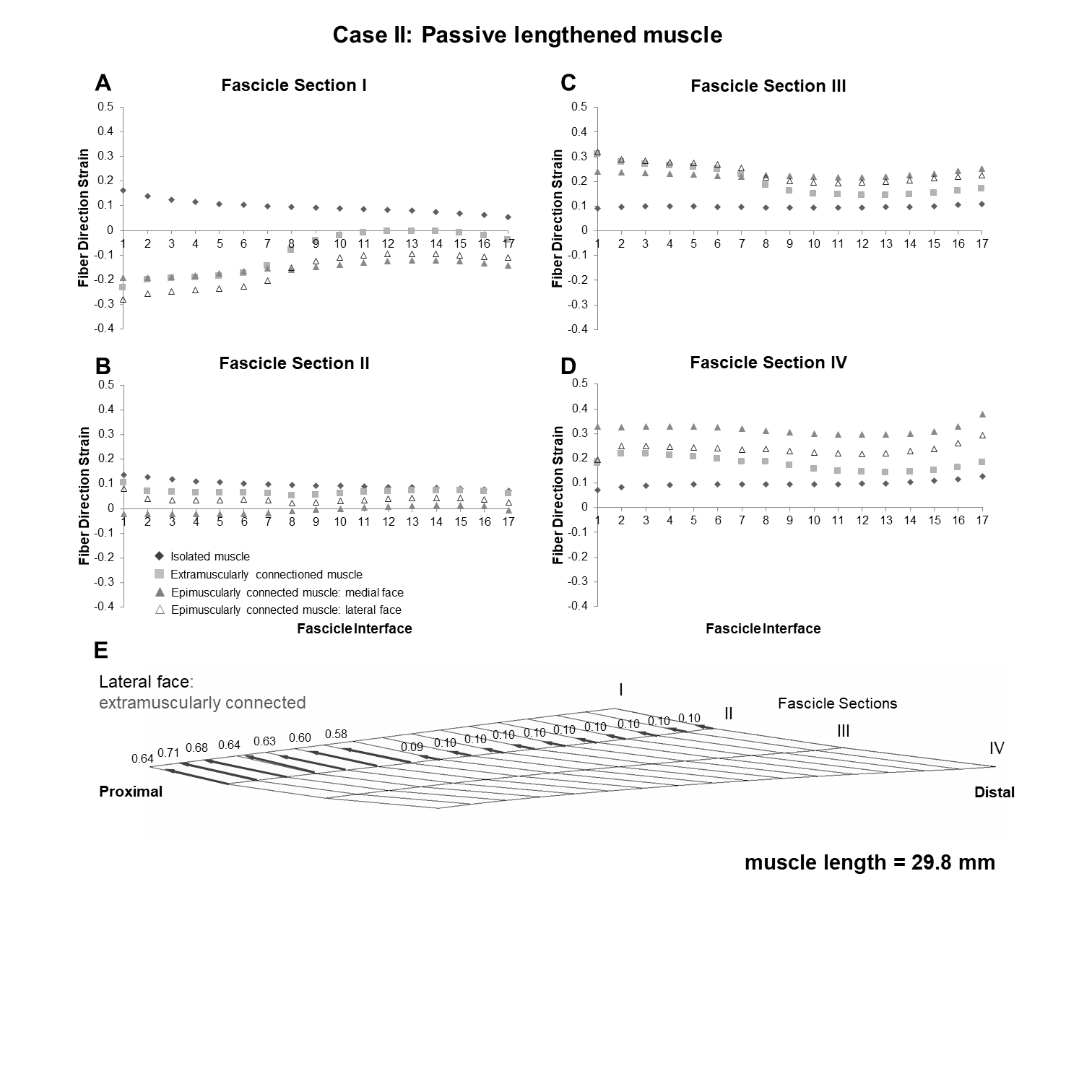


**Figure S7.** Fiber direction strains for passive target muscle at 29.8 mm length are plotted per fascicle sections I-IV **(A-D)** over fascicle interfaces 1-17 for isolated muscle, muscle with extra-muscular connections alone, and for medial and lateral faces of muscle with inter- and extra-muscular connections. **(E)** Extramuscular myofascial loads are depicted proportionately with glyphs on the lateral face of the target muscle.

*Case II: Passive lengthened muscle (to muscle length 29.5 mm)*

Isolated lengthened muscle shows lengthening in all fascicle interfaces, for all fascicle sections in muscle fiber direction (maximally by 11.8, 9.9, 7.8 and 9.2% in fascicle sections I, II, III and IV, respectively). However, the extramuscularly connected muscle does show in addition to lengthening occurring in most parts of the muscle, also shortening in muscle fiber direction (fascicle section I, up to 26.2% (*vs. 12.7% in main text*). This shortening effect is more pronounced in the epimuscularly connected muscle. Shortening in muscle fiber direction is shown in all fascicle interfaces of fascicle section I (maximally by 30.3% (*vs. 19.5%*) in the lateral face). Note that, myofascial loads acting on the extra- and epimuscularly connected muscles also limit (fascicle sections: I and II, maximally by 30.3% shortening and 7.3% lengthening, respectively) (Fig. S8 A-B) and elevate (fascicle sections: III and IV, maximally by 29.4 and 26.3%, respectively) lengthening in the remainder nodes (Fig. S8 C-D).

Normalized epimuscular loads on the lateral face of epimuscularly connected muscle range between 0.50 and 0.64 (*vs. 0.83 and 1 in main text*) for the stiffer proximal nodes, and are in excess of 0.08 (*vs. 0.13*) for remainder less stiff connections (Fig. S8 E). Again, compared to the results shown in the main text, reduced imposed muscle lengthening yields lower amplitudes of epimuscular myofascial loads, because they are stretched less due to less pronounced relative position change of the target muscle. Yet, because also the imposed muscle length itself causes less pronounced lengthening in the muscle fiber direction, these loads locally can overcome that and lead to a more pronounced local shortening effect compared to those shown in the main text.


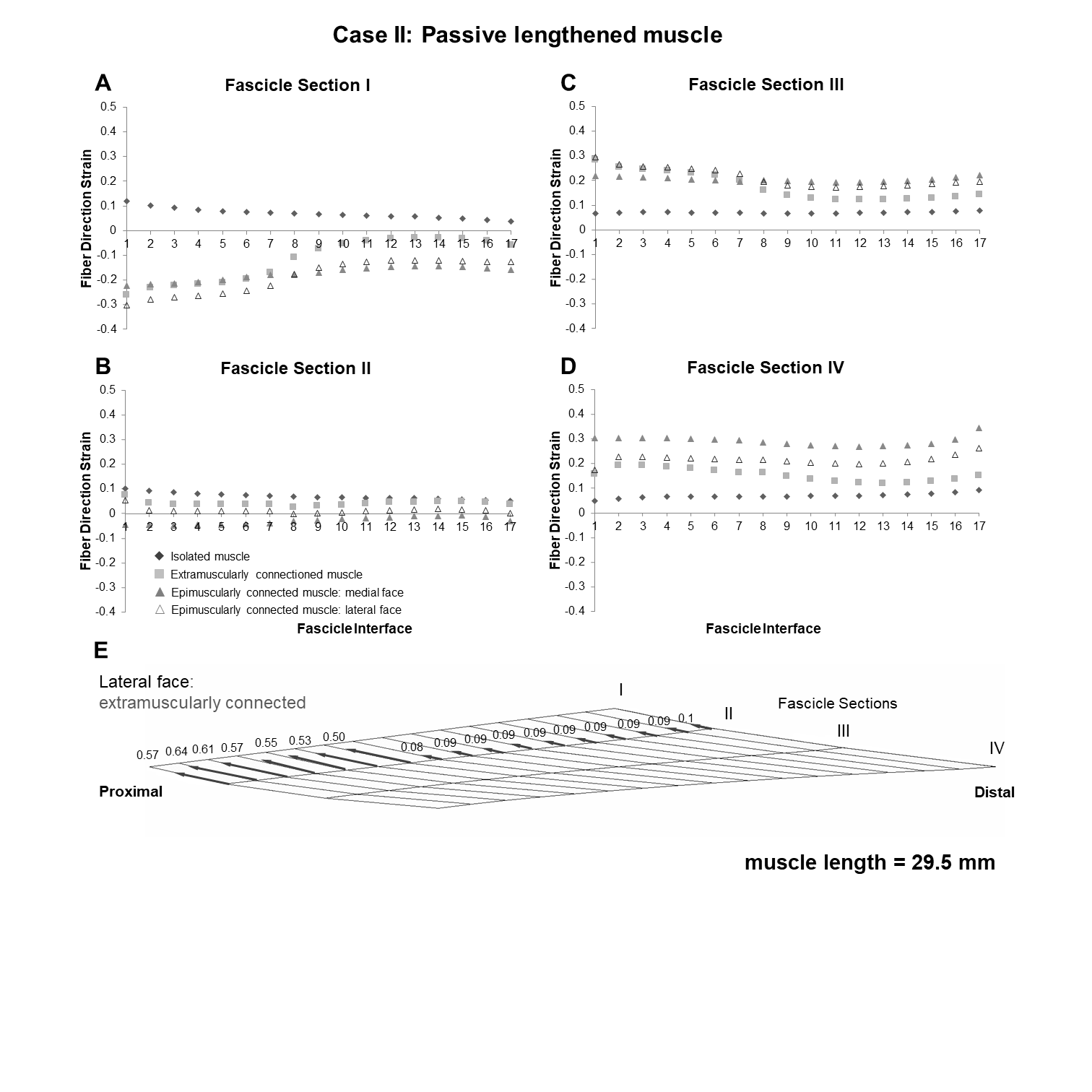


**Figure S8.** Fiber direction strains for passive target muscle at 29.5 mm length are plotted per fascicle sections I-IV **(A-D)** over fascicle interfaces 1-17 for isolated muscle, muscle with extra-muscular connections alone, and for medial and lateral faces of muscle with inter- and extra-muscular connections. **(E)** Extramuscular myofascial loads are depicted proportionately with glyphs on the lateral face of the target muscle.
